# Double artificial insemination in sheep: a comprehensive review

**DOI:** 10.1590/1984-3143-AR2024-0055

**Published:** 2025-05-19

**Authors:** Gabriel Maggi, Fabiane Pereira de Moraes, Fernando Caetano de Oliveira, Sergio Farias Vargas, Arnaldo Diniz Vieira, Rafael Gianella Mondadori, Bernardo Garziera Gasperin

**Affiliations:** 1 Faculdade de Veterinária, Universidade Federal de Pelotas – UFPel, Capão do Leão, RS, Brasil; 2 Faculdade de Veterinária, Universidade Federal do Rio Grande do Sul – UFRGS, Porto Alegre, RS, Brasil

**Keywords:** artificial insemination, sheep, cervical insemination, reproductive efficiency

## Abstract

Artificial insemination (AI) in sheep presents variable results, especially when combined with estrus induction treatments during the anestrous period. Alternatives for obtaining better results without significantly altering the costs of hormonal protocols are essential because of the importance of this biotechnology in production systems. One alternative that potentially meets these requirements is double-AI. Therefore, this article aims to review the literature on double-cervical AI in sheep and identify gaps in existing knowledge. Double cervical superficial (CS) AI with frozen-thawed (F.T.) semen after estrus detection significantly increased pregnancy rates (PR) in most (6/8) evaluated studies, with an increase of 7 to 34.2 percentual points (p.p.), compared to single AI. Regarding fixed-time AI (FTAI), all studies used fresh (F) or chilled (C) semen, and no positive effects were observed for double FTAI in most cases (8/9). Most studies have not applied current estrous synchronization protocols and insemination doses. Therefore, further studies are needed to evaluate the potential benefits of double FTAI, especially using F.T. semen in combination with hormonal protocols and insemination doses aligned with current practices.

## Introduction

Artificial insemination (AI) is an important reproductive biotechnique for ruminant production systems and an excellent tool for producers seeking genetic improvements in their herds (Alvarez et al., 2019). This technique can be combined with hormonal treatments to synchronize estrus and ovulation, which allows for fixed-time artificial insemination (FTAI) (Abecia et al., 2012).

The use of hormonal treatments for cyclicity induction and AI has several advantages, such as the resumption of cyclicity in sheep’s seasonal anestrus, programming of mating and lambing periods, and providing adequate climatic and nutritional conditions for ewes and their offspring. Additionally, treatment with equine chorionic gonadotropin (eCG) can increase the ovulation rate and the number of lambs (Menchaca and Rubianes, 2004).

AI in sheep presents variable conception rates, which are mainly related to the difficulty in depositing semen in the uterus owing to the tortuosity of the cervical canal (Fair et al., 2019). Laparoscopic insemination, an expensive technique for intrauterine semen deposition, is typically recommended for high-value frozen semen (Alvarez et al., 2019). In sheep farming, cervical superficial (CS) insemination is the primary method, wherein semen is deposited to traverse the cervical canal to the uterine lumen. The CS route is mainly indicated for AI with fresh or cooled-stored semen and can be performed after estrus detection or at a fixed time (Gibbons et al., 2019).

Although the CS AI is a simple, quick, and low-cost procedure, the results can vary significantly. Factors such as nutritional deficiencies, low body condition scores, poor semen quality, and animal management can compromise the results. Furthermore, inadequate hormonal protocols and asynchronous ovulations can negatively affect conception rates (Abecia et al., 2012; Gibbons et al., 2019).

A potential strategy to increase pregnancy rates obtained with CS AI is to perform double insemination, which is based on the deposition of two doses of semen in the female reproductive tract within a short period to increase the likelihood of fertilization. Asynchronous ovulation negatively affects fertility obtained through AI techniques, as the semen deposited in the female reproductive tract gradually loses viability over time. Thus, a second AI would be beneficial because it could provide viable sperm closer to ovulation (Salamon et al., 1979). Therefore, this review aims to report and discuss the data present in the literature on the application of double CS AI in sheep, identifying gaps in existing knowledge about this practice.

## Double cervical superficial artificial insemination

Double AI in sheep can be performed after estrus detection or after estrus and ovulation synchronization treatments using progestogens, eCG, and prostaglandin F2 alpha (PGF) analogs. In addition, AI can be performed at different intervals ranging from 3 to 24 h between inseminations. Such variations can affect reproductive efficiency and will be discussed in detail in this review.

### Double artificial insemination after estrus detection

The use of double AI has been reported since the early 1970s (Table 1). Salamon and Lightfoot (1970) were the first to report double insemination in ewes. Their findings indicated an increased lambing rate among ewes subjected to this technique using CS (38.8 vs. 22.6%). The insemination interval using frozen semen was approximately 12 h. Interestingly, the authors reported an acceptable lambing rate (LR; 39.7%) after a single AI with a reduced number of sperm cells (80x10^6^).

**Table 1:** Reproductive results obtained from single or double insemination in sheep based on the detection of estrus.

**Author**	**Hormone Treatment**	**Semen**	**Ins. Dose** **(x10^6^)**	**Double AI (h)**				**Results (%)**			
				**Single**	**1^st^AI**	**2^nd^ AI**	**Int. (h)**	**Single AI**	**Double AI**	**≠ (p.p)**	**P-value**
				**AI (h)**							
Salamon and Lightfoot (1970)•	Natural estrus	F.T.	150	1-15	1-15	13-27	12	22.6 (31/137)	38.8 (50/129)	16.2	< 0.01
	Natural estrus	F.T.	80	1-15	1-15	13-27	12	39.7 (46/116)	53 (61/115)	13.3	< 0.05
Salamon (1971)•	N.E. or 2nd post IVD	F.T.	150	1-2	1-15	10-25	10	42.9 (33/77)	59.5 (47/79)	16.6	overall < 0.02
	N.E. or 2nd post IVD	F	150	1-2	1-15	10-25	10	69.1 (47/68)	78.6 (55/70)	10.5	
	N.E. or 2nd post IVD	F.T.	150	1-2	1-2	11-12	10	40.9 (38/93)	55.1 (54/98)	14.2	overall < 0.02
	N.E. or 2nd post IVD	F.T.	150	1-2	1-2	11-12	10	39.1 (36/92)	50.5 (48/95)	11.4	
Visser and Salamon (1973)•	2nd post IVD	F.T.$	180	1-15	1-15	8-23	8	22.9 (8/35)	57.1 (20/35)	34.2	overall < 0.001
	2nd post IVD	F.T.^$^	180	1-15	1-15	8-23	8	35.3 (12/34)	54.5 (18/33)	19.2	
	2nd post IVD	F	180	1-15	1-15	8-23	8	54.3 (19/35)	77.1 (27/35)	22.8	
Visser and Salamon (1974)•	2nd post IVD	F.T.	90	12-14	12-14	23-25	11	19.4 (7/36)	35.3 (12/34)	15.9	overall < 0.01
	2nd post IVD	F.T.	180	12-14	12-14	23-25	11	29.7 (11/37)	57.6 (19/33)	27.9	
	2nd post IVD	F.T.	90	12-14	12-14	23-25	11	30.8 (12/39)	37.1 (13/35)	7.0	
	2nd post IVD	F.T.	180	12-14	12-14	23-25	11	34.2 (13/38)	54.5 (18/33)	20.3	
Salamon (1977)•	2nd post IVD	F.T.	90 vs. 45+45	12-14	12-14	23-25	11	31.1 (14/45)	34.1 (15/44)	3.0	
	2nd post IVD	F.T.	180 vs. 90+90	12-14	12-14	23-25	11	46.5 (20/43)	48.9 (22/45)	2.4	NS
	2nd post IVD	F.T.	360 vs. 180+180	12-14	12-14	23-25	11	55.6 (25/45)	55.8 (24/43)	0.2	
Salamon et al. (1979)•	2nd post IVD	C	150	12-14	12-14	24-26	12	34.6 (72/208)	55.2 (112/203)	20.6	< 0.001
Muñoz et al. (2002)◊	MPA (12d)	F.T.	200	18	3	6	3	20.5	22.0	1.5	NS
	MPA (12d)	F.T.	200	18	6	12	6	20.5	31.8	11.3	
	MPA (12d)	F.T.	200	18	12	18	5	20.5	21.7	1.2	
Simonetti et al. (2002)•	MPA (14d) + eCG(400IU)	F	300	7-11	3-4	-	8 - 9	64.4 (67/104)	67.7 (42/62)	3.0	> 0.05
Paulenz et al. (2003)•	Natural estrus	C	150	12-24	12-24	-	24	56.8 (150/264)	61.6 (183/297)	4.8	= 0.06
Jha et al. (2020) ^◊^	2x PGF	F.T.	200	12-18	10-12	16-18	6	16.7 (4/24)	26.1 (6/23)	9.4	> 0.05
	2x PGF	F.T.	100	12-18	10-12	16-18	6	0 (0/18)	12.5 (3/24)	12.5	
Palacios et al. (2022)*	Natural estrus	C	200	5-16	5-16	29-40	24	34 (8/23)	43 (6/14)	9.0	overall = 0.6
	Natural estrus	C	200	< 5	< 5	< 29	25	37 (4/11)	40 (4/10)	3.0	
	Natural estrus	C	200	16-24	16-24	40-48	24	42 (9/21)	42 (5/12)	0.0	

AI: artificial insemination; p.p.: percentual points; N.E.: natural estrus; MAP: medroxyprogesterone acetate; IVD: intravaginal device; eCG: equine chorionic gonadotropin; PGF: prostaglandin F2α; IU: international units. Symbols in the author’s column: •Studies that evaluated lambing rate; *Studies that evaluated fertility rate; ◊Studies that evaluated pregnancy rate. ^$^Different diluents, no diluent effect. Semen: fresh (F); chilled (C); or frozen-thawed (F.T.). Inseminating dose (x10^6^) of motile sperm. The time intervals correspond to the moments of insemination after estrus detection.

In a study with a similar experimental design, Salamon (1971), the LR in ewes subjected to two CS AI with an interval of 10 h was higher than that in the group that received a single AI, regardless of the use of fresh or frozen semen. Visser and Salamon (1973) evaluated different diluents using F.T. or fresh semen. They observed an increase in the LR of ewes subjected to double CS AI (with an interval of 8 h between AI) compared to the group that received a single AI. However, in both studies, the authors used a high number of sperm cells (150x10^6^). Therefore, performing two AI would likely be unfeasible under commercial conditions.

Visser and Salamon (1974) hypothesized that the benefits of double AI were associated with the greater number of spermatozoa deposited in the female reproductive tract. They observed that a single AI with a higher number of spermatozoa at the inseminating dose also led to an increase in lambing rates. For instance, when inseminating doses containing the same total number of cells for both single and double AI (180 × 10^6^ or 90 + 90 × 10^6^ motile spermatozoa) were compared, there was no difference in the LR, suggesting that the increase in reproductive rates observed in other studies was related to a greater number of sperm cells (Salamon, 1977). In agreement with this hypothesis, Muñoz et al. (2002) using F.T. semen after estrus detection did not observe a significant increase in the PR after double AI, which may be explained by the high number of cells (200 × 10^6^) used in each AI.

It is important to consider that these studies were performed during the 70’s decade and used semen frozen in pellets. Jha et al. (2020) performed estrus detection after a synchronization protocol using two doses of a PGF analog. They did not observe any differences in the PR of ewes inseminated with single or double AI (insemination interval: 6h). AIs were performed via the CS route using the same total number of motile spermatozoa (200 × 10^6^ in Study 1 and 100 × 10^6^ in Study 2). This study used a small number of animals (18 to 24 ewes per group) and no significant difference was observed, even with a numerical increase of 9.4 and 12.5 p.p. in pregnancy rate after double AI.

Other hypotheses have been proposed to explain the increase in LR after double AI. Salamon et al. (1979) suggested that the second insemination occurred more synchronously with the time of ovulation. In fact, an increase (20.6% p.p.) was observed in the LR of ewes inseminated twice with cooled-stored semen compared with ewes subjected to a single AI. Corroborating this finding, Paulenz et al. (2003) reported an increase in lambing rate in ewes inseminated twice with cooled-stored semen.

Palacios et al. (2022) proposed that double insemination using chilled semen could be a viable alternative to enhance pregnancy rates in sheep raised in organic farming systems, where hormonal treatments are prohibited. CS AI was performed in naturally cycling ewes at three different moments: 5 h, 5-16 h, and 16-24 h after estrus detection. After 24h, the ewes were inseminated again, and no increase in PR was observed in any group. This study has limitations: a reduced sample size (range, 10–23) and insemination with a higher sperm concentration (200 × 10^6^). Therefore, these limitations must be considered when evaluating the feasibility of double AI.

Simonetti et al. (2002), using progesterone and eCG for estrus synchronization, followed by estrus detection, did not observe differences in lambing rates between ewes inseminated once (7–11 h after estrus detection) or twice (3–4 h after estrus detection and 8–9 h after the first insemination). The authors reported high PR (64.4 and 67.7%), but unfortunately, they used an extremely high number of sperm cells (300 × 10^6^) in each AI, which may explain the absence of a significant increase in PR after double AI.

Most studies that performed double AI after estrus detection demonstrated increased pregnancy or lambing rates, especially in studies using F. T. semen. Although double CS AI could be considered an alternative to laparoscopy, studies evaluating the possibility of reducing the number of cells in CS dose insemination should be performed. Furthermore, the intensification of sheep farming, the search for a reduction in the number of animals handling and the need to keep rams or teasers on the property make estrus detection a limiting factor for AI applications. Thus, studies seeking to improve estrus and ovulation synchronization protocols are essential for disseminating other biotechniques. Double FTAI could represent an alternative for synchronized ewes, as it does not require estrus detection and reduces the number of semen collection and insemination procedures. It is also a low-cost alternative for increasing herd reproductive efficiency.

### Double artificial insemination at a fixed time

The success of a FTAI protocol depends mainly on the proper control of the time of ovulation and AI. Therefore, it seems logical that performing two FTAIs could bring benefits by increasing the chances of fertilization. In a study using ewes synchronized with two applications of PGF analogs, double FTAI increased the PR compared to ewes that received a single AI (60 or 66 h). However, there was no benefit from AI performed at 56 h (Table 2; Acritopoulou-Fourcroy et al., 1982). These results suggest that the positive effect of double AI depends on the timing of ovulation(s), which is difficult to predict, especially when ovulation inducers are not used. Therefore, performing a second AI could represent a viable alternative to ensure less variation in the PR when conducting PGF-based FTAI.

**Table 2:** Reproductive results obtained after single or double FTAI in sheep.

**Author**	**Hormone Treatment**	**Sem.**	**Ins. Dose (x10^6^)**	**Double AI (h)**				**Results Obtained (%)**			
				**Single AI (h)**	**1^a^ AI**	**2^a^ AI**	**AI Int. (h)**	**Single AI**	**Double AI**	**≠ (p.p)**	**P-value**
Smith et al. (1978)•	FGA + eCG (500UI)	C	400	56	48	58	10	71.7 (66/92)	60.2 (56/93)	-11.5	< 0.01
Acritopoulou-Fourcroy et al. (1982)•	2x PFG	F	160	56	56	66	10	54.8 (23/42)	61.9 (26/42)	7.10	> 0.05
		F	160	60	56	66	10	37.5 (12/32)	61.9 (26/42)	24.4	< 0.05
		F	160	66	56	66	10	30.8 (12/39)	61.9 (26/42)	31.1	< 0.05
Langford (1982)•	FGA (12d) + eCG(500IU)	C	450	54	54	60	6	67.0 (14/21)	61.0 (11/18)	-6.0	
		C	450	57	54	60	6	67.0 (12/18)	61.0 (11/18)	-6.0	> 0.05
		C	450	60	54	60	6	37.0 (7/19)	61.0 (11/18)	24.0	
	FGA (12d)	C	450	54	54	60	6	11.0 (2/18)	33.0 (7/21)	22.0	
		C	450	57	54	60	6	11.0 (2/18)	33.0 (7/21)	22.0	> 0.05
		C	450	60	54	60	6	26.0 (5/19)	33.0 (7/21)	7.0	
Langford et al. (1982)•	FGA (12d) + eCG(500UI)	C	450	54	54	59	5	55 (26/47)	56 (30/54)	1.0	
		C	450	55	55	60	5	73 (35/48)	69 (34/49)	-4.0	> 0.05
		C	450	56	56	60	4	67 (31/46)	76 (34/45)	9.0	
Langford et al. (1983)•	30mg FGA (12d) + eCG(500IU)	C	450	55	55	60	5	52 (11/21)	70 (14/20)	18.0	> 0.05
	30mg FGA (12d)	C	450	55	55	60	5	10 (2/20)	11 (2/19)	1.0	> 0.05
	40mg FGA (12d) + eCG(500IU)	C	450	55	55	60	5	69 (9/13)	80 (8/10)	11.0	> 0.05
	40mg FGA (12d)	C	450	55	55	60	5	31 (4/13)	20 (2/10)	-11.0	> 0.05
Langford (1986)•	30mg FGA (14d) + eCG(500IU)	C	450	55	55	60	5	39 (37/96)	27 (27/99)	-12	> 0.05
	40mg FGA (12d) + eCG(500IU)	C	450	55	55	60	5	68 (79/117)	69 (29/42)	1	
Fukui et al. (1991)•	MPA (9d) + eCG(600IU)	F	500	36	30	42	12	44.6 (25/56)	50.9 (29/57)	6.3	> 0.05
	500mg P4 (9d) + eCG(600IU)	F	500	36	30	42	12	46.6 (27/58)	50 (30/60)	3.4	> 0.05
Menchaca et al. (2005)◊	MPA (6d)+ PGF+eCG (250IU)	C	200	48	48	54	6	34.7 (17/49)	23.4 (11/47)	-11.3	< 0.05
		C	200	54	48	54	6	10.6 (5/47)	23.4 (11/47)	12.8	< 0.05
Burutaran et al. (2024) ^◊^	2x PFG (15d)	F	150	54	44	68	24	66.4 (83/125)	73.8 (93/126)	7.4	> 0.05
		F	150	44	44	68	24	64.1 (82/128)	73.8 (93/126)	9.7	> 0.05
		F	150	68	44	68	24	66.7 (84/126)	73.8 (93/126)	7.1	> 0.05

AI: artificial insemination; p.p.: percentual points; MPA: medroxyprogesterone acetate; FGA: fluorogestone acetate; eCG: equine chorionic gonadotropin; PGF: prostaglandin F2α; IU: international units. Symbols in the authors column: •Studies that evaluated lambing rate; *Studies that evaluated conception rate; ◊ studies that evaluated pregnancy rate. Semen (Sem.): fresh (F); chilled (C). Inseminating dose (x10^6^) of motile sperm.

A study using double administration of PGF and fresh semen (150 × 10^6^ motile spermatozoa) did not observe advantages in the use of double FTAI (44 and 68 h after the second PGF) compared to single AI (44, 54 or 68 h after the second PGF; Burutaran et al., 2024). A high PR was obtained with both single (64.1 to 66.7%) and double (73.8%) FTAI.

Double AI did not increase the LR in animals subjected to long-duration progestogen-based protocols (12-14 days), with or without eCG (500 IU), and using chilled semen (450 × 10^6^ motile spermatozoa) (Langford, 1982, 1986; Langford et al., 1982, 1983). Notably, these studies used an inseminating dose with a total number of cells four times higher than the conventionally used dose; therefore, the eventual benefits of the two FTAIs may have been underestimated.

Fukui et al. (1991) used protocols with medroxyprogesterone acetate (MPA) or progesterone IVDs for nine days, associated with eCG (600 IU), and observed no difference between single and double insemination, with an interval of 12 h. Again, the high number of cells used in AI does not match current practices.

Menchaca et al. (2005) evaluated the association of double insemination with short-duration P4 protocols, with PGF at IVD insertion and eCG (250 IU) at device removal and did not observe a difference in the pregnancy rate at 35 days, using an interval of 6 h between inseminations (48 and 54 h after IVD removal). The pregnancy rates in the study were lower (10-35%) than expected (35-60%), regardless of treatment, suggesting that other variables may have interfered with the results. Some authors have also reported lower pregnancy rates in ewes subjected to double FTAI, attributing this result to the possible impact of the stress caused by additional handling (Smith et al., 1978). However, only two of the nine studies reported a negative effect of double FTAI on PR.

These studies indicated no benefits of applying double FTAI, especially when high PR was obtained in the single FTAI group. Considering the need for extra labor and animal handling of both ewes and rams, double FTAI was not indicated. None of the studies in the literature have used ovulation inducers such as hCG or GnRH. Therefore, the additional use of ovulation inducers may increase the chances of fertilization after performing one or two FTAIs. Better control of the time of ovulation can reduce the number of sperm cells at insemination doses. However, these hypotheses still need to be tested.

## Conclusion

The results reported in the literature regarding double CS AI after estrus detection and double FTAI are contradictory. It was observed that double CS AI using F.T. semen after estrus detection significantly increased the PR in most studies. No positive effects of double FTAI have been reported in most studies. Therefore, further studies are necessary to increase the potential benefits of double FTAI, especially when F.T. semen is used with hormonal protocols and AI procedures aligned with current practices. Furthermore, future research should investigate the factors contributing to the inconsistent pregnancy rates reported in the literature, such as the interval between inseminations, the use of ovulation inducers, and the impact of additional handling on animal stress.

## Data Availability

No research data was used.

## References

[B001] Abecia JA, Forcada F, González-Bulnes A (2012). Hormonal control of reproduction in small ruminants. Anim Reprod Sci.

[B002] Acritopoulou-Fourcroy S, Pappas V, Peclaris G, Zervas N (1982). Synchronization of oestrus in ewes with Provera sponges/PMSG, prostaglandin F2alpha or the prostaglandin analogue, ICI 80996, and fertility following natural mating or artificial insemination. Reprod Nutr Dev.

[B003] Alvarez M, Anel-Lopez L, Boixo JC, Chamorro C, Neila-Montero M, Montes-Garrido R, Paz P, Anel L (2019). Current challenges in sheep artificial insemination: A particular insight. Reprod Domest Anim.

[B004] Burutaran M, Fierro S, Negrín F, Minteguiaga M, Gil J, Olivera–Muzante J (2024). Estrous, ovulation and reproductive responses of ewes synchronized with a long interval prostaglandin–based protocol for timed AI. Theriogenology.

[B005] Fair S, Meade KG, Reynaud K, Druart X, De Graaf SP (2019). The biological mechanisms regulating sperm selection by the ovine cervix. Reproduction.

[B006] Fukui Y, Yamamoto Y, Goda S, Ono H (1991). Single or double inseminations at fixed-time basis on lambing rate of ewes treated with progestogen-impregnated intravaginal sponges during the non-breeding season. Jpn J Anim Reprod..

[B007] Gibbons AE, Fernandez J, Bruno-Galarraga MM, Spinelli MV, Cueto MI (2019). Technical recommendations for artificial insemination in sheep. Anim Reprod.

[B008] Jha PK, Alam GS, Al Mansur A, Talukder MRI, Naher N, Anisur Rahman AKM, Hall DC, Bari FY (2020). Effects of number of frozen-thawed ram sperm and number of inseminations on fertility in synchronized ewes under field condition. J Anim Reprod Biotechnol..

[B009] Langford GA (1986). Influence of body weight and number of Iinseminations on fertility of progestogen-treated ewe lambs raised in controlled environments. J Anim Sci.

[B010] Langford GA (1982). Influence of PMSG and time of artificial insemination on fertility of progestogen-treated sheep in confinement. J Anim Sci.

[B011] Langford GA, Ainsworth L, Wolynetz MS (1982). Reproductive response of progestatogen-treated sheep in confinement to a single and double insemination. J Anim Sci.

[B012] Langford GA, Marcus GJ, Batra TR (1983). Seasonal effects of PMSG and number of inseminations on fertility of progestogen-treated sheep. J Anim Sci.

[B013] Menchaca A, Pinczak A, Queirolo D (2005). Storage of ram semen at 5 °C: effects of preservation period and timed artificial insemination on pregnancy rate in ewes. Anim Reprod.

[B014] Menchaca A, Rubianes E (2004). New treatments associated with timed artificial insemination in small ruminants. Reprod Fertil Dev.

[B015] Muñoz C, Parraguez VH, Latorre E (2002). Efecto del tiempo de inseminación artificial después de la detección de celo sobre la tasa de preñez en ovinos corriedale. Agric Téc.

[B016] Palacios C, Abecia JA, Plaza J, Hidalgo C, de la Fuente LF (2022). Efficiency of artificial insemination at natural estrus in organic churra ewes. Vet Sci.

[B017] Paulenz H, Söderquist L, Ådnøy T, Fossen OH, Berg KA (2003). Effect of milk- and TRIS-based extenders on the fertility of sheep inseminated vaginally once or twice with liquid semen. Theriogenology.

[B018] Salamon S (1977). Fertility following deposition of equal numbers of frozen-thawed ram spermatazoa by single and double insemination. Aust J Agric Res.

[B019] Salamon S (1971). Fertility of ram spermatozoa following pellet freezing on dry ice at -79 and -140°C. Aust J Biol Sci.

[B020] Salamon S, Lightfoot RJ (1970). Fertility of ram spermatozoa frozen by the pellet method III. The effects of insemination technique, oxytocin and relaxin on lambing. J Reprod Fertil.

[B021] Salamon S, Maxwell W, Firth J (1979). Fertility of ram semen after storage at 5 °C. Anim Reprod Sci.

[B022] Simonetti L, Ramos G, Gardón JC (2002). Effect of estrus synchronization and artificial insemination on reproductive performance of Merino sheep. Braz J Vet Res Anim Sci.

[B023] Smith PA, Boland MP, Gordon I (1978). Conception rate in ewes: effect of method of breeding and number of inseminations. J Agric Sci.

[B024] Visser D, Salamon S (1974). Fertility following Inseminations with Frozen-Thawed Reconcentrated and Unconcentrated Ram Semen. Aust J Biol Sci.

[B025] Visser D, Salamon S (1973). Fertility of ram spermatozoa frozen in a tris-based diluent. Aust J Biol Sci.

